# Cardiometabolic risk variables in overweight and obese children: a worldwide comparison

**DOI:** 10.1186/1475-2840-10-106

**Published:** 2011-11-24

**Authors:** Mariska van Vliet, Martijn W Heymans, Inès A von Rosenstiel, Desiderius PM Brandjes, Jos H Beijnen, Michaela Diamant

**Affiliations:** 1 Department of Pediatrics, Slotervaart Hospital, Louwesweg 6, 1066 EC, Amsterdam, the Netherlands; 2 Department of Internal Medicine, Slotervaart Hospital, Louwesweg 6, 1066 EC, Amsterdam, the Netherlands; 3 Department of Pharmacy & Pharmacology, Slotervaart Hospital, Louwesweg 6, 1066 EC, Amsterdam, the Netherlands; 4 Diabetes Center, Department of Internal Medicine, VU University Medical Center, De Boelelaan 1118, 1081 HV, Amsterdam, the Netherlands; 5 Department of Biostatistics, VU University Medical Center, De Boelelaan 1118, 1081 HV, Amsterdam, the Netherlands

## Abstract

The growing prevalence rate of pediatric obesity, which is frequently accompanied by several cardiometabolic risk factors, has become a serious global health issue. To date, little is known regarding differences for cardiometabolic risk factors (prevalence and means) in children from different countries. In the present review, we aimed to provide a review for the available evidence regarding cardiometabolic risk factors in overweight pediatric populations. We therefore provided information with respect to the prevalence of impaired fasting glucose/impaired glucose tolerance, high triglycerides, low HDL-cholesterol and hypertension (components of the metabolic syndrome) among cohorts from different countries. Moreover, we aimed to compare the means of glucose and lipid levels (triglycerides and HDL-cholesterol) and systolic/diastolic blood pressure values. After careful selection of articles describing cohorts with comparable age and sex, it was shown that both prevalence rates and mean values of cardiometabolic risk factors varied largely among cohorts of overweight children. After ranking for high/low means for each cardiometabolic risk parameter, Dutch-Turkish children and children from Turkey, Hungary, Greece, Germany and Poland were in the tertile with the most unfavorable risk factor profile overall. In contrast, cohorts from Norway, Japan, Belgium, France and the Dominican Republic were in the tertile with most favorable risk profile. These results should be taken with caution, given the heterogeneity of the relatively small, mostly clinical cohorts and the lack of information concerning the influence of the values of risk parameters on true cardiometabolic outcome measures in comparable cohorts. The results of our review present a fair estimation of the true differences between cardiometabolic risk profiles among pediatric cohorts worldwide, based on available literature.

## Introduction

The ongoing global rise in the prevalence of overweight and obesity among all age and ethnic groups is accompanied by a higher incidence in serious health risks, such as type 2 diabetes (T2DM), and the development of cardiovascular damage, which is already potentially present at an early age.[1-3] In both children and adults, detection of the metabolic syndrome, which encloses a clustering of cardiometabolic disorders (i.e. central obesity, disorders in glucose regulation, dyslipidemia and hypertension) is a tool to identify individuals with high risk on future T2DM and cardiovascular disease (CVD).[4-6] However, no validated definition with standardized cut-off values of the metabolic syndrome is available in children, and as a consequence, many derivatives from adult the metabolic syndrome-definitions are used to attempt to implement the metabolic syndrome in pediatrics.[7-10] Therefore, a wide range of prevalence rates of the metabolic syndrome among pediatric cohorts has been reported, making it impossible to draw conclusions with respect to differences between cohorts from different countries. To overcome this issue in the present review, description and comparison of the prevalence of single cardiometabolic risk factors (the metabolic syndrome-criteria; impaired fasting glucose (IFG)/impaired glucose tolerance (IGT), low HDL-cholesterol, high triglycerides and hypertension) was performed.

Although it is difficult to standardize data from different studies regarding age and BMI, we embarked on this study to identify potential early trends with respect to cardiometabolic variables between children from different countries. The premise of this review was to estimate CVD risk by the number of the metabolic syndrome features, assuming that the risk for future CVD conferred by the metabolic syndrome in adults is proportioned to the number of the metabolic syndrome features present. Moreover, mean values of aforementioned variables between different countries can be compared and used to estimate cardiometabolic risk. In the present review, we performed a systematic literature search, and aimed to provide an overview of available studies reporting data regarding cardiometabolic risk factors in pediatric cohorts with different ethnicities and nationalities.

## Methods

### Prevalence/mean values of cardiometabolic risk factors

**Figure 1:**
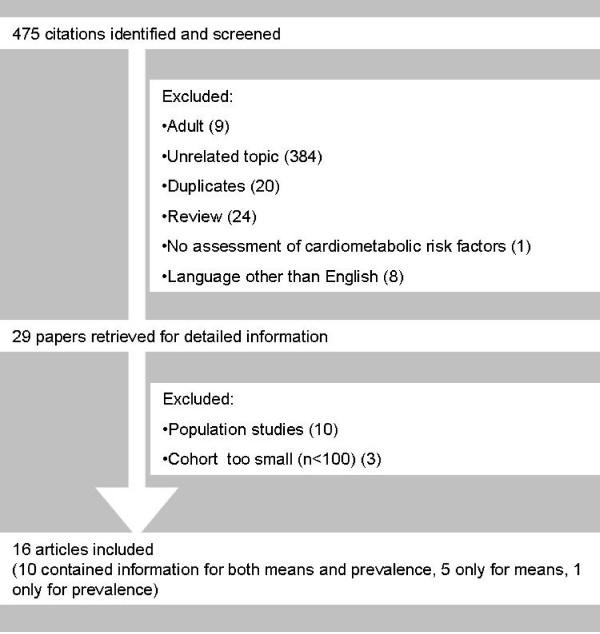
**Literature search**.

Information was collected regarding the prevalence and means of the features of the metabolic syndrome (impaired fasting glucose (IFG), impaired glucose tolerance (IGT), low HDL-cholesterol, high triglycerides and high blood pressure).[9] Therefore, we searched PubMed, EMBASE and the COCHRANE library for studies investigating the metabolic syndrome in youth using the MeSH terms: 'Overweight', 'Obesity', 'Metabolic Syndrome X', 'Prevalence', with limitation to studies in humans which have been published in English and in an age range of 0-18 years. Figure 1a depicts the flowchart showing the study inclusion steps. One independent reviewer (MvV) selected all studies which included more than 50 children (in a wider age range, i.e. the cohort had to consist of both prepubertal and pubertal children) and which reported information on multiple outcome measures, being reported in either mean values or prevalence numbers.

### Data extraction

Figure 1 shows the literature search, which was carried out from April to August 2009. This search was performed by the first author and a clinical librarian, specialised in systematic search strategies. The selection procedure was performed by the first author. Studies which were classified as non-eligible were studies with unrelated topics or in languages other than English, studies among older age groups (>20 years), reviews, and studies that did not assess (components of) the metabolic syndrome. After retrieval of 29 possible eligible studies, 13 more were excluded due to study design (population studies) or small cohorts (n<100), and a total of 16 studies could be included in the present review.

### Assessment of prevalence numbers

To overcome the use of different cut-off values for the cardiometabolic risk factors, we derived new prevalence rates (high triglycerides above the 95^th ^percentile for age and sex (>p95), low HDL-cholesterol <1.03 mmol/L and hypertension >p95) from the reported prevalence rates. This was performed with use of a reference group, from which the distribution of prevalence rates for different cut-off values was derived. The reference group consisted of the data from Dutch native children from our own multi-ethnic cohort (3-18 yrs, n = 262), and was compared with the distribution of the prevalence rates from the other ethnic groups. The formentioned cut-off values were chosen in order to minimize the number of estimated values. Ratios between prevalence numbers from the old and new cut-off values in the reference population were used to estimate the prevalence in the cohort of interest. Since the distribution of prevalence rates using different cut-off values was equal over all ethnic groups within the reference cohort, it was assumed that this also applied for the other study cohorts.

### Assessment of mean values and statistical analysis

In thirteen papers, data for means and standard deviations (SD's) with regard to cardiometabolic parameters were available. In three articles, one or more parameters were reported as median (range) or median (interquartile range, IQR). When median values (range, or IQR) were provided, the SD was estimated according to a previously validated method, in which the mean and variance were estimated using simple formulas which included the range and median.[11] To obtain differences between other populations and the Dutch reference population, Students t-tests were applied, after estimating the standard error of the mean difference for each comparison (using the SD and N), as recently described.[12] Differences between different ethnic groups as derived from the reference population were tested with ANOVA and Bonferoni analysis (Dutch-Turkish, Dutch-Moroccan and Dutch-Other groups).

### Ranking

In order to produce a rough estimation of overall cardiometabolic risk profile for each study cohort, mean values of each country were ranked from highest to lowest presumed cardiometabolic risk (or from lowest to highest in case of HDL-cholesterol). Subsequently, an overall rank was adopted from these separate rank scores and the outcome was divided into tertiles. Since it was found that elevation of systolic blood pressure predicts the metabolic syndrome and hypertension in later life best (with consequently higher risk for T2DM and CVD), we decided to include only the systolic blood pressure ranks in the analysis.[13]

## Results

### Characteristics of included studies

Characteristics of studies are shown in Table 1. Most studies were performed in a clinical setting, however, four were identified as population studies, but could be included in the analysis due to a sufficient number and separate analysis of overweight children. All studies were cross-sectional in nature.

**Table 1:** Study characteristics of included studies for comparison of prevalence numbers/means

Country	Author^Ref^	N	Patient characteristics	Study nature	Data	BMI (kg/m^2^)/Z-BMI
The Netherlands	Van Vliet et al.[34]	95	Dutch native, Turkish, Moroccan and Other subgroups, 3-18y	Clinical	Prevalence/mean	27.7 ± 5.9/2.8 ± 0.5

Italy^1^	Invitti et al.[27]	588	6-16y	Clinical	Prevalence/mean	33.9 ± 5.5

France	Druet et al.[36]	308	7-17y	Clinical	Prevalence/mean	28 (24.4-32.3)

Spain	Lopez-Capape et al.[37]	429	4-18y Caucasian and Hispanic	Clinical	Prevalence/mean	27.3 ± 3.5

Turkey^1^	Atabek et al.[38]	169	7-18y	Clinical	Prevalence/mean	27.2 ± 3.6

Turkey^2^	Sen et al.[20]	352	2-19y	Clinical	Prevalence/mean	29.1 ± 4.3

USA	Dhuper et al.[18]	576	3-19y, mainly black cohort	Population	Prevalence/mean	33.2 ± 7.6

USA	Davis et al.[39]	102	7-18y, rural Georgia, black and white mixed	Population	Prevalence/mean	27.6 ± 5.2

Dominican Republic	Sherry et al.[40]	193	2-20y	Clinical	Prevalence/mean	31.3/2.6

Germany	Reinehr et al.[19]	463	4-17y	Clinical?	Prevalence/mean	27.1 (24.5-29.9)/2.36

Central Europe (Germany/Switserland/Austria)	Allemand et al.[14]	25473	1-20y	Clinical	Prevalence	>29.4/2.32

Norway	Kolsgaard et al.[33]	120	Norwegian, native subgroup, 6-17y	Clinical?	mean	28.1 (25.5-31.0)/2.1 ± 0.3

China	Li et al.[41]	620	7-18 yrs	Population	mean	>p85

Belgium	Beauloye et al.[16]	104	8-18 yrs	Clinical	mean	30.2 ± 0.5/2.8 ± 0.7

Japan	Islam et al.[17]	471	7-15y	Annual health examination	Mean	25.7 ± 2.4

			4.5-18.2y			25.7 ± 2.4
Poland, Greece, Italy^2^, France, Hungary	Bokor et al.[42]	90	Polish	Clinical	mean	30.8 ± 4.9
		145	Greek			30.1 ± 4.9
		274	Italian			26.8 ± 3.6
		283	French			37.7 ± 5.9
		449	Hungarian			31.1 ± 4.8

Data regarding BMI and Z-BMI are shown as mean ± SD or median (interquartile range)

Both studies by Allemand et al.[14] (Central Europe) and Li et al. [15] (China) provided no information with regard to methods of measuring height and blood pressure. All other studies reported resting blood pressure values or average of three separate measurements while seated. Information regarding the method of height measurements (i.e. use of stadiometer) were lacking in the studies by Bealoye et al. [16] (Belgium), Islam et al. [17] (Japan), Dhuper et al. [18] (USA), Reinehr et al. [19] (Germany) and Sen et al. [20] (Turkey).

### Variation of prevalence rates across countries

**Figure 2:**
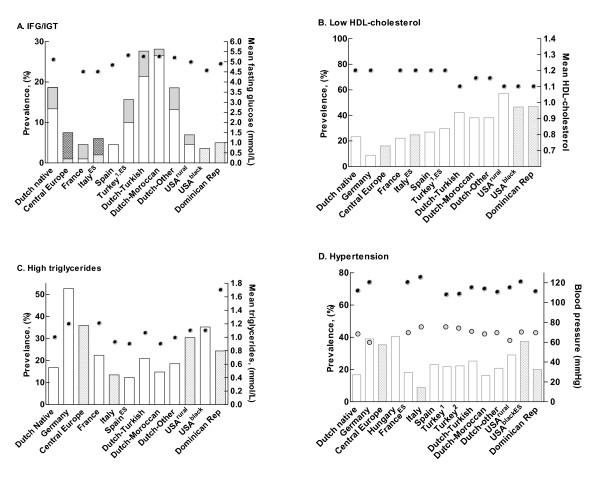
**Prevalence rates of cardiometabolic risk factors according to country**. IFG is indicated by light grey bars, IGT by dark grey bars. Blocked bars indicate a cohort with a BMI >30 kg/m^2^. ^ES^Estimated prevalence from distribution in reference cohort. USA^rural ^Cohort from the USA from rural area (both black and white children included). USA^black ^Cohort from the USA with predominantly black children. *IFG-impaired fasting glucose, IGT-impaired glucose tolerance, Dominican Rep-Dominican Republic*.

Figure 2 shows the prevalence of cardiometabolic risk factors according to different countries. Whereas the prevalence of impaired fasting glucose (≥5.6 mmol/L) was very low among pediatric cohorts in Southern European countries (1.0 to 4.6%), the USA (3.6-4.6%) and Dominican republic (5%), the prevalence rate in the Netherlands (especially among Dutch-Turkish and-Moroccan children) was remarkably high; 13.4% and 26.8%, respectively. IGT varied from 1.3% (Dutch-Moroccan children) to 6.5% in Central Europe. Similar prevalence rates for IGT were found in Turkish children and Dutch-Turkish children. Regarding lipid profiles, children from Germany least frequently showed low HDL-cholesterol (8.8%), while a high percentage of children from rural USA presented with this lipid anomaly (57%). High triglycerides were most prevalent in Germany (52.7%) whereas prevalence rates varied in the other countries from 12.3% to 35.9%. Finally, the presence of elevated blood pressure varied from 8.8% in Italy, to 40.6% among children from Hungary.

### Variation of mean values of cardiometabolic risk parameters

**Figure 3:**
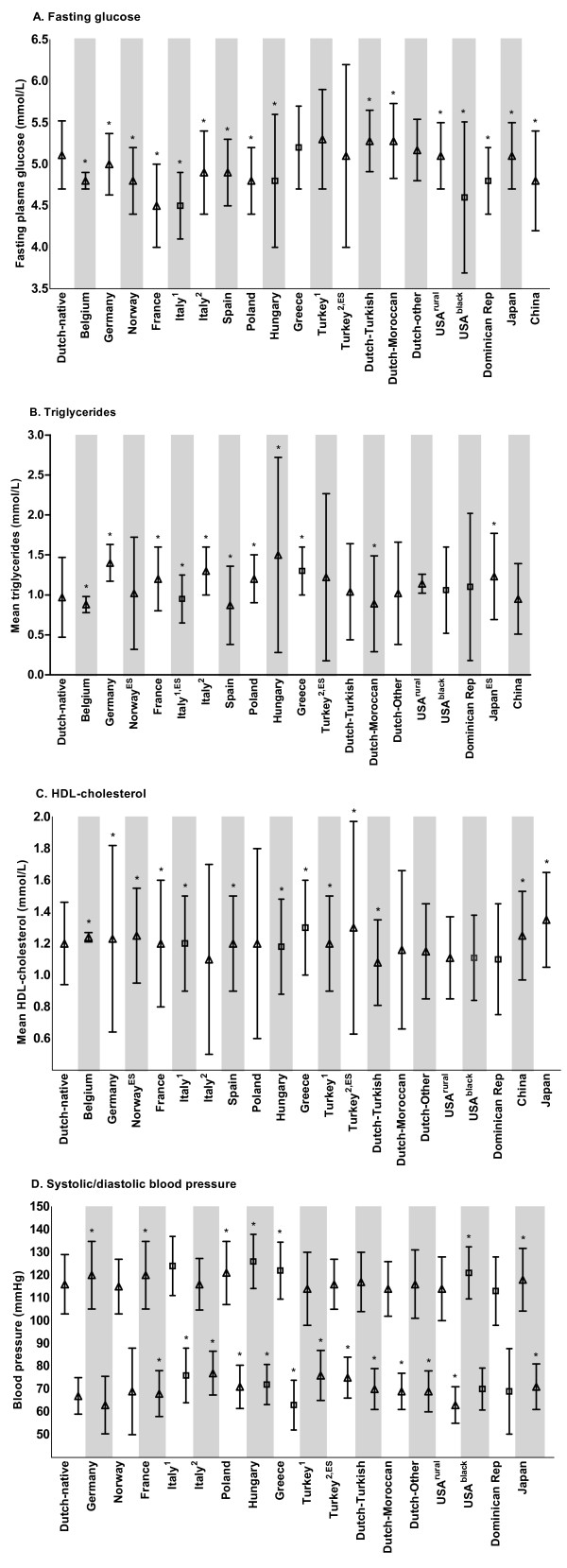
**(continued) Mean values of cardiometabolic risk variables according to country**. Data are shown as mean ± SD across countries. Means displayed as a triangle indicate cohorts with a mean BMI between 25 and 30 kg/m^2^. Means displayed as squares indicate cohorts with a mean BMI>30 kg/m^2^. For blood pressure, both systolic (upper means and whiskers) and diastolic (lower means and whiskers) are shown. *P<0.05 as compared to Dutch native children. ^ES^Mean and SD estimated from median (range or interquartile range). *Dominican Rep - Dominican Republic*.

Figure 3 shows a large variation for mean values of the cardiometabolic variables per country. Fasting glucose levels varied from 4.5 to 5.3 mmol/L (in France and Turkey, respectively). The mean value of HDL-cholesterol ranged from 1.1 mmol/L in Dutch-Turkish children, and cohorts from the USA, Italy and Dominican republic, to 1.4 mmol/L in Japan. Triglyceride levels were highest in Hungary and Turkey (respectively 1.5 and 1.6 mmol/L), while mean BMI of these cohorts did not exceed 30 kg/m^2^. Systolic blood pressure was generally highest in countries from which cohorts exceeded 30 kg/m^2^, with a value of 121 mmHg (Poland) to 126 mmHg (Hungary). Children in the Dominican Republic showed the lowest mean value of systolic blood pressure, despite their mean BMI of 34 kg/m^2^.

### Overall cardiometabolic risk

Table 2 shows the ranking of countries per cardiometabolic parameter and overall cardiometabolic risk profile rank. Children from Hungary, Greece, Germany, and Poland, as well as Dutch-Turkish children were in the tertile with the most unfavorable cardiometabolic risk profile. In contrast, children from Norway, China, Belgium, France and Dominican Republic together made up the tertile with the presumably most favorable cardiometabolic risk profile, while the mean BMI of the cohort in Belgium and the Dominican Republic still exceeded 30 kg/m^2^. Contradictive results were derived from the two studies from Italy and two studies from Turkey, from which one cohort in each country was classified as having a favorable cardiometabolic risk profile, while the other was assigned an unfavorable cardiometabolic risk rank.

**Table 2:** Ranking countries for most adverse cardiometabolic risk factor profile

Country (ref)	Mean BMI (kg/m^2^)	Mean glucose rank	Mean triglyceride rank	Mean HDL-cholesterol rank	Mean systolic blood pressure rank	Total cardiometabolic risk rank
Hungary [42]	31.1	13	2	8	1	**1**

Dutch-Turkish [34]	29.3	2	13	1	9	**2**

Turkey^1 ^[38]	27.2	1	1	9	15	**3**

Italy^2 ^[42]	26.8	8	4	2	10	**4**

Greece [42]	30.1	4	4	18	3	**5**

Germany [19]	27.1	10	3	14	6	**6**

Poland [42]	30.8	13	8	9	4	**7**

Dutch-Other [34]	29.1	5	14	6	10	**8**

USA rural [39]	27.6	7	10	4	15	**9**

USA black [18]	33.2	19	12	4	4	**10**

Japan [17]	25.7	7	6	20	8	**11**

Dutch-native [34]	28.7	6	16	9	10	**11**

Turkey^2 ^[20]	29.1	7	7	18	10	**13**

Spain [37]	27.3	11	21	9	-	**13-19**

Dutch-Moroccan	28.4	2	19	7	15	**14**

France [36]	37.7	20	8	9	6	**14**

Dominican Republic [40]	34	13	11	2	18	**16**

Italy^1 ^[27]	33.9	20	17	9	2	**17**

China [41]	-	13	17	16	-	**17-19**

Norway [33]	28.2	13	14	16	14	**18**

Belgium [16]	30.2	13	20	15	-	**18-19**

Ranking was performed according from highest to lowest mean value for each cardiometabolic risk parameter (lowest to highest in case of HDL-cholesterol). Secondly, an overall mean rank was assigned to each cohort

## Discussion

The present review showed a large variation for prevalence rates and mean values of cardiometabolic risk parameters among children according to country of origin, and reveals several interesting findings. In summary, children from Norway, China, Belgium, France and the Dominican Republic constitute the tertile with the most favorable values of cardiometabolic risk parameters. In contrast, children from Central and Eastern Europe (Germany and Poland), together with South Eastern Europe (Hungary, Greece) and Turkey constitute the third of children with the most adverse values of cardiometabolic risk parameters. The former is in line with the finding that mortality rates from CVD are generally higher in Central and Eastern Europe than in Northern, Southern and Western Europe, as concluded by a report concerning cardiovascular disease statistics.[21]

Some of the comparisons between cohorts from different countries have been studied before, however, most studies originate from the United States, with the focus on differences between the white, black and Hispanic population. Therefore, the cardiometabolic differences between black and white children living in the USA are well-known (i.e. a more favorable lipid profile and slightly higher prevalence of hypertension.[7,22-26] Our review supports this finding, and adds that this difference is also found between black children and other cohorts, however, children from Greece and Hungary present with similar blood pressure values as the cohort of children of mainly African-American descent. Although some differences may be explained by characteristics of the cohort (i.e. more obese), others cannot. In example, high triglycerides and high blood pressure were quite low in an Italian, extremely obese cohort, while they were frequently present in a moderately obese German cohort, and in a cohort from Central Europe (Germany/Switzerland/Austria).[14,27] Therefore, it is presumed that genetic profile and dietary habits play a substantial role outside the degree of obesity in affecting cardiometabolic risk parameters.

The relation between mean values in relation to prevalence rates remains under debate. Despite varying mean values amongst populations from different origins or ethnic backgrounds, reference values suggested by international guidelines are often applied, assuming a linear relationship between mean values of cardiometabolic parameters and actual cardiometabolic risk. However, it has been shown that not all populations have similar baseline risk for CVD and therefore, ethnic-specific reference values are warranted. To illustrate, in Mexico, an unusual high prevalence of low HDL-cholesterol among non-overweight subjects was found, (NCEP reference values), namely 83%.[28] Surprisingly, mortality from CVD among the Mexican population is lower than among non-Hispanic whites.[29] Therefore, one might argue that lower HDL-cholesterol levels among Hispanics (as compared to whites) are acceptable and do not lead to a higher mortality rate. In contrast, one might hypothesize that interventions improving lipid metabolism may lead to an even lower prevalence of death from CVD.

A recent review reported a lower prevalence of lipid disorders in black children as compared to white children, and were less frequently diagnosed with the metabolic syndrome as a consequence.[30] This finding is contradictory to the finding that blacks are having more frequently diabetes and CVD as compared to whites. To overcome this discrepancy, the review suggested that lipid reference values in black children should be more strict, in order to achieve a prevalence of the metabolic syndrome which would correspond better with actual cardiometabolic outcome. Although the adjustment of lipid levels reference values might in part accomplish forementioned goal, probably hypertension and other factors such as ox-LDL, hs-CRP and IL-6 (which are not accounted for in the metabolic syndrome) may cause the higher incidence of diabetes and CVD among blacks.[31]^,^[32] Therefore, in case of African-American children, the use of stricter cut-off values for lipid levels will probably not induce identification of those most at risk for CVD. Further studies are needed to determine the optimal reference values for cardiometabolic risk parameters in specific populations.

Also within countries, large differences between means for cardiometabolic risk parameters were found, which limits extension of the outcome to the general population. In example, cohorts from the two studies from Italy (Milano, mean BMI 33.9 ± 5.5 and Rome, mean BMI 26.8 ± 3.6, respectively), showed opposite cardiometabolic characteristics. While the cohort from Milano (despite the higher mean BMI), was in the tertile with the most favorable cardiometabolic risk profile, the cohort from Rome was in the terile with the most adverse cardiometabolic risk profile. Selection bias and geographical location of both institutions (ie outskirts vs. city center) may have accounted for the difference in outcomes.

The difference between cardiometabolic risk profile between the Turkish cohorts may be explained by differences in age range (2-19 vs. 7-18 yrs), confirming a more adverse cardiometabolic risk profile in older children. Interestingly, Dutch-Turkish children, (age range 3-18 yrs), presented with a similar risk profile as the older cohort (7-18 yrs) from Turkey. Forementioned findings thus indicate that Dutch-Turkish children have a cardiometabolic risk profile which is even less favorable as compared to children living in Turkey.

Next to age and geographical location, varying BMI may account for some of the differences found between the studies. It would be expected that the cardiometabolic risk parameters would be more favorable in similar samples with lower BMI values. Although, in the present review, the former statement was true for blood pressure (highest among extremely obese subjects), this did not apply to glucose levels, as they were lowest among the extremely obese cohorts. Since we do not have data from moderately obese children from the same country, the meaning of this finding remains unclear, however it might be hypothesised that in some cohorts, a favorable genetic profile may preponderate over increasing BMI in glucose regulation.

Few studies have been performed with regard to cardiometabolic risk factors among children of ethnic descent within countries. In a Norwegian study which compared immigrants (Pakistani, Tamil and Turkish) to native Norwegian children, higher rates of insulin resistance and the metabolic syndrome were found among immigrant subjects.[33] In a study performed among 516 multi-ethnic children in the Netherlands, the cardiometabolic risk profile appeared more favorable in children of Moroccan descent, and more adverse in children of Turkish origin, as compared to Dutch native children. This previous finding is partly confirmed by the results of the present study, in which Turkish cohorts appear to have high mean glucose and triglyceride levels (but also high HDL-cholesterol).[34] It may be postulated that immigration of Turkish children to the Netherlands has an additional adverse effect on cardiometabolic risk profile. This may be attributed to several reasons, among which change of dietary habits and less physical exercise.

One of the limitations of this review is the inclusion of mainly studies of clinical nature, with small sample sizes, which makes it hard to extend the individual study outcomes to the general population of a specific country. Despite careful selection of the studies, there was still some heterogeneity among them, and small differences between methods of blood pressure measurements and measuring height could have in part attributed to some the differences found. Moreover, data with respect to the values of cardiometabolic parameters in childhood and the actual cardiometabolic risk in adulthood are scarce. A single study was performed using mortality as primary end-point, and reported that glucose intolerance, and hypertension (not high total cholesterol) in childhood were strongly associated with increased rates of premature death from endogenous causes.[35] Due to aformentioned reasons, the outcome of this review should be interpreted with caution; however, we believe that this review gives some insight and a good overview of available studies on the subject of cardiometabolic risk in childhood obesity.

In conclusion, among pediatric cohorts, consisting of both pubertal and prepubertal children, a wide variation of mean values for different cardiometabolic parameters were found, with most favorable values in cohorts of children from Norway, China, Belgium, France and the Dominican Republic, and least favorable values in a cohort of Dutch-Turkish children, as well as children from cohorts from Germany, Poland, Hungary, Greece and Turkey. These results should be taken with caution, given the heterogeneity of the relatively small, mostly clinical cohorts and the lack of information concerning the influence of the values of risk parameters on true cardiometabolic outcome measures in comparable cohorts.

## Competing interests

The authors declare that they have no competing interests

## Authors' contributions

MvV wrote the first draft of the manuscript. MvV and MD contributed in concept and design, data collection, analysis and interpretation, drafting the article and revision. MwH contributed in the concept and design, analysis and interpretation of data, and revision. IvR, DPMB and JB contributed to the design, data collection, interpretation of data, and revision. All authors have read and approved the final manuscript.
